# Telmisartan mitigates lipopolysaccharide (LPS)-induced production of mucin 5AC (MUC5AC) through increasing suppressor of cytokine signaling 1 (SOCS1)

**DOI:** 10.1080/21655979.2021.1943605

**Published:** 2021-07-19

**Authors:** Ling Chen, Jiajia Xu, Meiyu Deng, Yanling Liang, Jinfu Ma, Linghui Zhang, Yijie Wang, Jinping Zhang

**Affiliations:** a Department of Respiration, No. 305 Hospital of PLA, Beijing, China; b Department of Pathology, Zhongda Hospital Southeast University, Nanjing, Jiangsu, 210009, China; c Department of Endocrinology, No. 305 Hospital of PLA, Beijing, 100017, China; d Department of Gerontology, No. 305 Hospital of PLA, Beijing, 100017, China

**Keywords:** Acute lung injury, Telmisartan, inflammation, mucin 5AC (MUC5AC), suppressors of cytokine signaling 1 (SOCS1)

## Abstract

Acute lung injury (ALI) is a serious clinical pulmonary disease. The pathogenesis of ALI is related to the excessive release of inflammatory factors and upregulation of mucin 5AC (MUC5AC). Telmisartan is a novel antihypertension agent that exerts promising anti-inflammatory effects. The purpose of this study is to investigate whether Telmisartan has a protective role in lipopolysaccharide (LPS)-induced MUC5AC expression and to explore the underlying mechanism in human bronchial epithelial cells. Firstly, the decreased cell viability, elevated release of lactate dehydrogenase (LDH), and excessively released inflammatory factors tumor necrosis factor-α (TNF-α), interleukin- 6 (IL-6), and transforming growth factor-β (TGF)-β in bronchial BEAS-2B epithelial cells induced by stimulation with LPS were significantly reversed by the introduction of Telmisartan. Secondly, the upregulated MUC5AC and downregulated suppressor of cytokine signaling 1 (SOCS1) caused by stimulation with LPS were dramatically reversed by Telmisartan. Notably, treatment with Telmisartan attenuated LPS-induced activation of nuclear factor κ-B (NF-κB). Lastly, silencing of SOCS1 abolished the protective effects of Telmisartan against LPS-induced production of MUC5AC and the activation of NF-κB. Based on these findings, we conclude that Telmisartan displayed a protective effect against LPS by improving mitochondrial function, mitigating inflammatory response, and reducing the production of mucin 5AC by regulating the SOCS1/NF-κB axis in human bronchial epithelial cells.

## Graphical abstract

**Figure:**
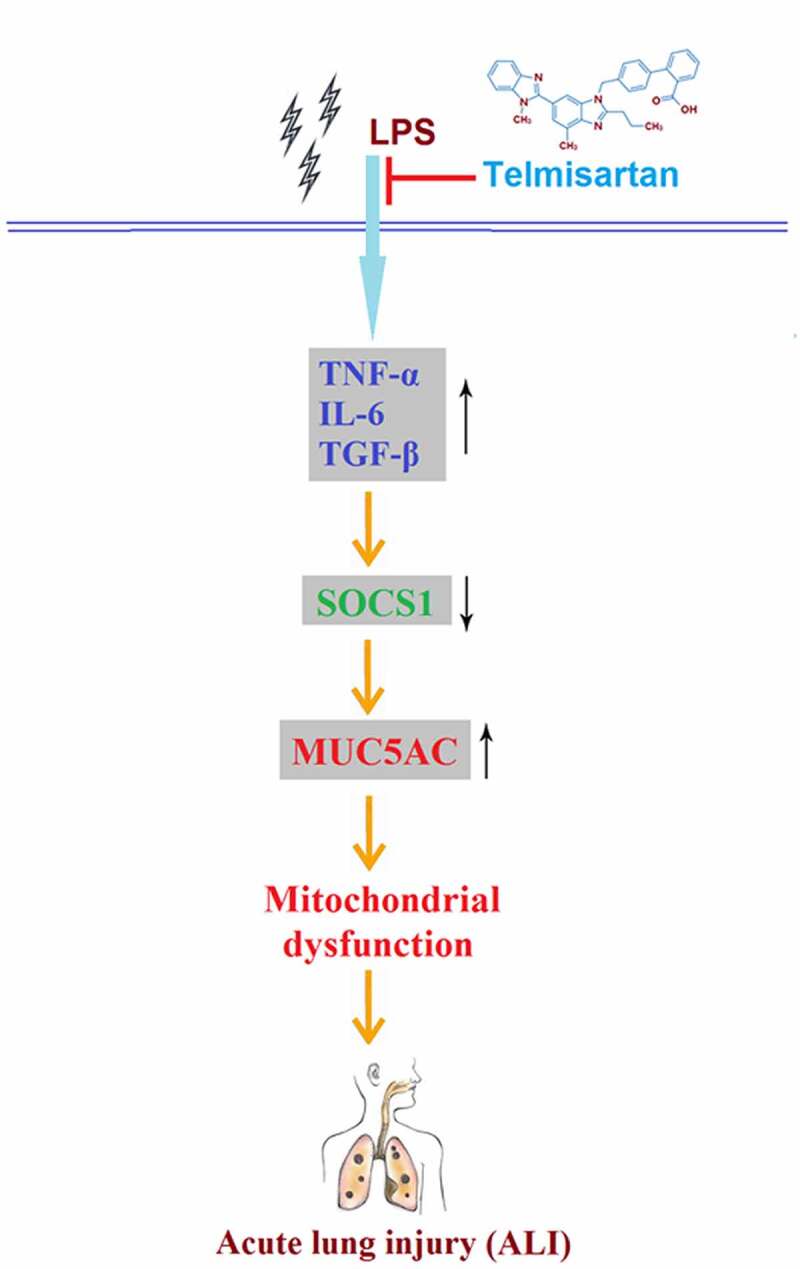

## Introduction

Acute lung injury (ALI) is clinically characterized by progressive hypoxemia and respiratory distress. It is regarded as the early stage of acute respiratory distress syndrome (ARDS), caused by indirect or direct pulmonary injury triggered by internal or external elements. As a common clinical critical disease, the mortality rate of ALI is approximately 30–40% [1,2]. So far, the prophylaxis and treatments of ALI remain very limited [3,4]. Therefore, it is urgent to investigate the pathogenesis and explore the new target for the treatment of clinical ALI. At present, the underlying pathological mechanism of ALI is unclear [5]. However, it is particular that the edema fluid, rich in protein, entering the alveoli, induced by severe inflammation, and vascular endothelial injury is involved in the development and processing of ALI [6].

Firstly, the monocytes/macrophages system is activated by stimulation with lipopolysaccharide (LPS), leading to the excessive release of inflammatory factors including interleukin-1β, tumor necrosis factor (TNF)-α, and interleukin- 6 (IL-6). These proinflammatory factors mediate the adhesion, migration, and chemotaxis of leukocytes by upregulating the expression of adhesion molecules and cytoselectins, which contribute to the accumulation of leukocytes in the lung tissues [7,8]. Following the adhesion between the leukocytes and the vascular endothelial cells, the coagulation system is activated. Excessive production of inflammatory factors (cytokines, lipid metabolism, leukotrienes, and proteases) indirectly or directly acts on the endothelial cells to impact the alveolar-capillary barrier function [9]. As a result, the integrity of pulmonary microvascular endothelium is disrupted, the permeability promoted, and the exudation of fluids and macromolecules in blood vessels induced, contributing to the pulmonary edema in the development of ALI [10,11]. Excessive secretion of respiratory mucus is one of the important characteristics of ALI, its main components include mucin, water, and some macromolecules [12]. According to reports, Mucin 5AC (MUC5AC), as the main component of respiratory secretions, can enhance the transport of neutrophils, thereby further aggravating the process of lung injury [13]. Therefore, inflammatory factors and MUC5AC might be promising targets for the treatment of ALI. Suppressors of cytokine signaling 1 (SOCS1) is a member of the SOCS family of proteins. SOCS1 suppresses cytokine-induced NF-κB signaling and is an important inhibitor in bronchial epithelial cells [14]. Previous studies have shown that SOCS1 is a negative regulator of MUC5AC expression. It suppresses LPS-induced MUC5AC hypersecretion in airway cells through inhibiting NF-κB [15,16].

**Figure 1.**
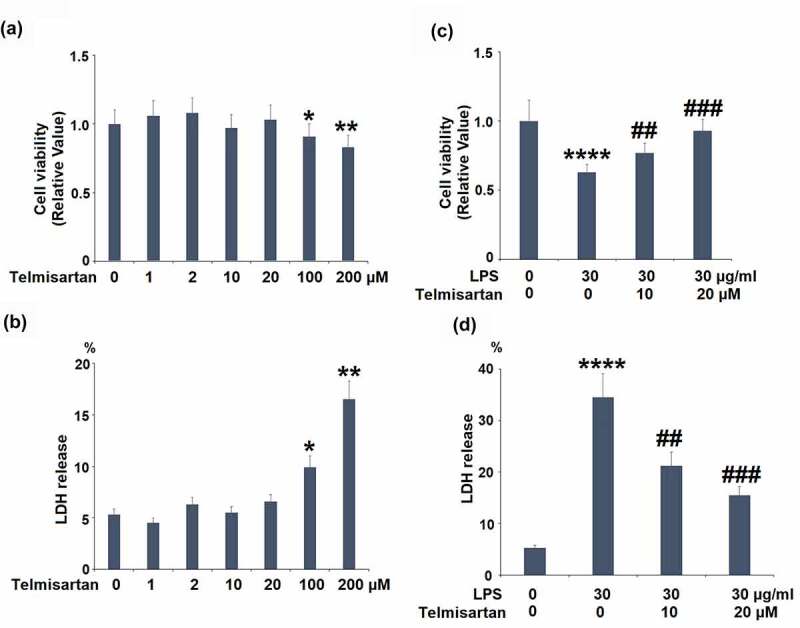
Cytotoxicity of Telmisartan in bronchial BEAS-2B epithelial cells. (a-b). Cells were incubated with Telmisartan at concentrations of 1, 2, 10, 20, 100, and 200 μM for 24 hours. Cell viability normalized to vehicle group while LDH release normalized to total LDH in each group (%); (c-d). Cells were challenged with LPS (30 μg/ml) with or without Telmisartan (10, and 20 μM) for 24 hours. Cell viability normalized to vehicle group while LDH release normalized to total LDH in each group (%) (*, **, ****, P < 0.05, 0.01, 0.0001 vs. vehicle group; ##, ###, P < 0.01, 0.001 vs. LPS group)

Telmisartan is a novel angiotensin II receptor 1 blocker (ARB) and partial agonist of peroxisome proliferators-activated receptor-γ (PPAR-γ) developed by Boehringer Ingelheim for the treatment of hypertension [17]. According to the results from clinical trials, apart from antihypertensive effects, Telmisartan (Figure 1) was found to alleviate insulin resistance and type II diabetes, suppress the expression of inflammatory factors, and prevent the development of inflammation [18]. Wu reported that in diabetic cardiomyopathy rats, the inflammatory response and myocardial apoptosis were significantly ameliorated by the administration of Telmisartan by regulating the transforming growth factor-β1 (TGF)-β1/smad signaling pathway [19]. In the present study, we aim to investigate the protective effects of Telmisartan against LPS-induced inflammatory response and the expression of MUC5AC in bronchial BEAS-2B epithelial cells, and to explore the potential molecular mechanism.

## Materials and methods

### Cell culture and treatments

The bronchial BEAS-2B epithelial cells were purchased from ATCC (Maryland, USA) and cultured in Dulbecco’s Modified Eagle Medium (DMEM) supplemented with 5% fetal bovine serum (FBS) at 37°C and 5% CO_2_. The treatment reagents LPS (Escherichia coli O127:B8) and Telmisartan (98% purity, HPLC grade) were purchased from Sigma-Aldrich (St. Louis, USA). The concentration of Telmisartan was optimized with a series of dose-response experiments for 3-(4,5)-dimethylthiahiazo (-z-y1)-3,5-di-phenytetrazoliumromide (MTT) and LDH experiments (0, 1, 2, 10, 20, 100, and 200 μM) for 24 hours. For all other experiments, cells were challenged with LPS (30 μg/ml) with or without Telmisartan (10, and 20 μM) for 24 hours.

### MTT assay

After the treatments, the cells were mixed with 200 μL of 1 mg/mL MTT and incubated for 4 hours at 37°C. Subsequently, the MTT solution was removed and replaced with 200 μL DMSO solution. Lastly, the absorbance at 570 nm of each well was detected by a microplate reader (PerkinElmer, Massachusetts, USA).

### LDH release assay

The cytotoxicity was assessed with a commercial LDH assay kit (Abcam, UK). In brief, after the treatments, the culture supernatants in the appropriate culture medium were collected and diluted. 100 μL cell culture supernatants were added to each well of 96-well plates to react with 100 μL reaction mixture for 30 minutes. The absorbance at 490 nm was recorded with a microplate reader (PerkinElmer, Massachusetts, USA).

### Mitochondrial membrane potential (MMP)

The treated bronchial BEAS-2B epithelial cells were incubated with 5 μM fluorescent dye rhodamine 123 (Thermo Fisher Scientific, USA) for 10 minutes in an incubator, followed by being washed with PBS buffer. Lastly, the fluorescence was determined using a fluorescent microscope at 488 nm.

### The level of cellular adenosine-triphosphate (ATP)

The treated bronchial BEAS-2B epithelial cells were treated with 0.1% (vol/vol) Triton X-100 to quantify the intracellular ATP level, which was detected using the Enliten ATP assay (Leiden, Netherlands) and a microplate Luminometer (Berthold, Pforzheim, Germany).

### Enzyme-linked immunosorbent assay (ELISA)

The concentrations of TNF-α, IL-6, TGF-β, and MUC5AC were detected with ELISA assay. Briefly, the cell media was collected by spinning to remove the cell debris, and the soluble protein was quantitated with a bicinchoninic acid (BCA) kit (Thermo Fisher Scientific, USA). Then the monoclonal antibodies (R&D, Wiesbaden, Germany) were coated on the microplates, followed by three times washing and blocking with 0.2% casein buffer. Subsequently, the supernatant of the treated cells was added to be incubated for 1 hour, followed by being added with a secondary streptavidin peroxidase-conjugated antibody (R&D, Wiesbaden, Germany). Finally, the absorbance at 450 nm was measured using a microplate reader (Thermo, Massachusetts, USA).

### RT-PCR assay

Firstly, the total RNA from the treated bronchial BEAS-2B epithelial cells was isolated using the RNA extraction kit (Takara, Tokyo, Japan) according to the manufacturer’s instructions. After quantifying the concentration of the isolated RNA using the NanoDrop spectrophotometer (Thermo, Massachusetts, USA), the RNA was transcribed into cDNA with specifically random RT primers. Subsequently, the PCR procedure was performed using the SYBR Premix Ex Taq TM (Takara, Tokyo, Japan). Lastly, the relative expression level of target genes was determined using the 2^−ΔΔCt^ method following normalization with the expression level of glyceraldehyde-3-phosphate dehydrogenase (GAPDH). The following primers were used in this study: TGF-β (F: 5ʹ-CCCAGCATCTGCAAAGCTC −3ʹ, R: 5ʹ- GTCAATGTACAGCTGCCGCA −3ʹ); IL-6 (F: 5ʹ- TGGTCTTTTGGAGTTTGAGGTA −3ʹ, R: 5ʹ- AGGTTTCTGACCAGAAGAAGGA-3ʹ); TNF-α (F: 5ʹ- CTCTTCTGCCTGCTGCACTTTG-3ʹ, R: 5ʹ- ATGGGCTACAGGCTTGTCACTC-3ʹ); MUC5AC (F: 5ʹ- CCACTGGTTCTATGGCAACACC-3ʹ, 5ʹ- GCCGAAGTCCAGGCTGTGCG-3ʹ); SOCS1 (5ʹ-GCATCCCTCTTAACCCGGTAC-3ʹ, 5ʹ- AAATGAAGCCAGAGACCCTCC-3ʹ); GAPDH (5ʹ- GAACATCATCCCTGCCTCTACT −3ʹ, 5ʹ- GTCTACATGGCAACTGTGAGGA −3ʹ).

### Western blotting assay

Firstly, the total proteins were extracted from the treated bronchial BEAS-2B epithelial cells using the radio immunoprecipitation assay (RIPA) buffer, further quantified utilizing a BCA kit (Thermo Fisher Scientific, USA). Subsequently, the proteins were loaded and separated using the sodium dodecyl sulfate polyacrylamide gel electrophoresis (SDS-PAGE), followed by being transferred to a polyvinylidene fluoride (PVDF) membrane (Millipore, MIT, USA). The membranes were then blocked by the 5% BSA, followed by being incubated with primary antibodies against SOCS1 (Cell Signal Technologies, #3950), and β-actin (Cell Signal Technologies, #3700) at 4°C overnight. After washing and incubating with the secondary antibody at room temperature for 2 hours, the blots were incubated with the electrochemiluminescence (ECL) reagents (Beyotime, Shanghai, China), followed by being exposed to Tanon 5200-multi (Tanon, Shanghai, China). Lastly, Image J software (NIH, USA) was used to quantify the relative expression of target proteins densitometry.

### Luciferase activity assay

Cells (2 × 10^4^) were seeded to each well in 24- well plates and transfected with 100 ng

NF-κB -dependent luciferase expression plasmid using the lipofectamine 3000 (Thermo Fisher Scientific, USA), followed by stimulation with LPS (30 μg/ml) or Telmisartan (10, 20 μM) for 24 hours. After that, cells were collected and lysed, luciferase activity was measured using the luciferase assay system (Promega). The results were normalized to protein concentrations.

### Small interfering RNA (siRNA) knockdown

The knockdown of SOCS1 in BEAS-2B was based on the commercial ON-TARGET plus siRNA source (Thermo Fisher Scientific, USA). In brief, 0.3 million BEAS-2B cells were plated in 60-mm dishes and were allowed to reach about 50% confluence. The next day, 50 nM of SOCS1 specific siRNA pool (or nonspecific control) was mixed with 6 μL Lipofectamine RNAi Max (Thermo Fisher Scientific, USA) and 400 µL of serum-free media to form the transfection delivery reagent. The reacted reagents were then added to antibiotic-free media and incubated for 4 hours. After 48–96 hours, the efficiency of knockdown was evaluated with Western blot.

## Statistical analysis

Data are shown as mean ± standard deviation (S.D.) of at least three independent experiments. Analysis of variance (ANOVA) was used for multiple group comparison. The main effects were then compared by a Newman-Keuls’ posthoc test. P < 0.01 was considered to be significant and indicated by asterisks.

## Results

In this study, using an LPS-challenged human bronchial epithelial cell model, we investigated the beneficial effects of Telmisartan against LPS. We report that Telmisartan mitigated LPS-induced mitochondrial dysfunction and the production of inflammatory cytokines. Importantly, we found that Telmisartan reduced MUC5AC expression through the SOCS1/NF-κB signaling pathway.

### Cytotoxicity of Telmisartan in bronchial BEAS-2B epithelial cells

To screen the optimized incubation concentration of Telmisartan in bronchial BEAS-2B epithelial cells, cells were incubated with Telmisartan at concentrations of 1, 2, 10, 20, 100, 200 μM for 24 hours, followed by being measured by MTT assay and LDH release. As shown in Figure 1a, as the concentration of Telmisartan increased from 1 to 20 μM, no significant difference was observed in the cell viability. However, as the concentration of Telmisartan was promoted to 100 or 200 μM, the cell viability was significantly declined. As shown in Figure 1b, as the concentration of Telmisartan increased from 1 to 20 μM, the release of LDH was maintained around 5%, which was dramatically elevated to 9.9% and 16.5% by the introduction of 100, and 200 μM Telmisartan. Therefore, in the subsequent experiments, the 10, and 20 μM Telmisartan were used.

We then investigated whether Telmisartan possessed a protective effect against LPS-induced reduction of cell viability and increased LDH release. Interestingly, we found that LPS stimulation reduced cell viability but increased LDH release in bronchial BEAS-2B epithelial cells, which was rescued by Telmisartan dose-dependently (Figure 1c and d).

### Telmisartan improved LPS-induced mitochondrial dysfunction in bronchial BEAS-2B epithelial cells

**Figure 2.**
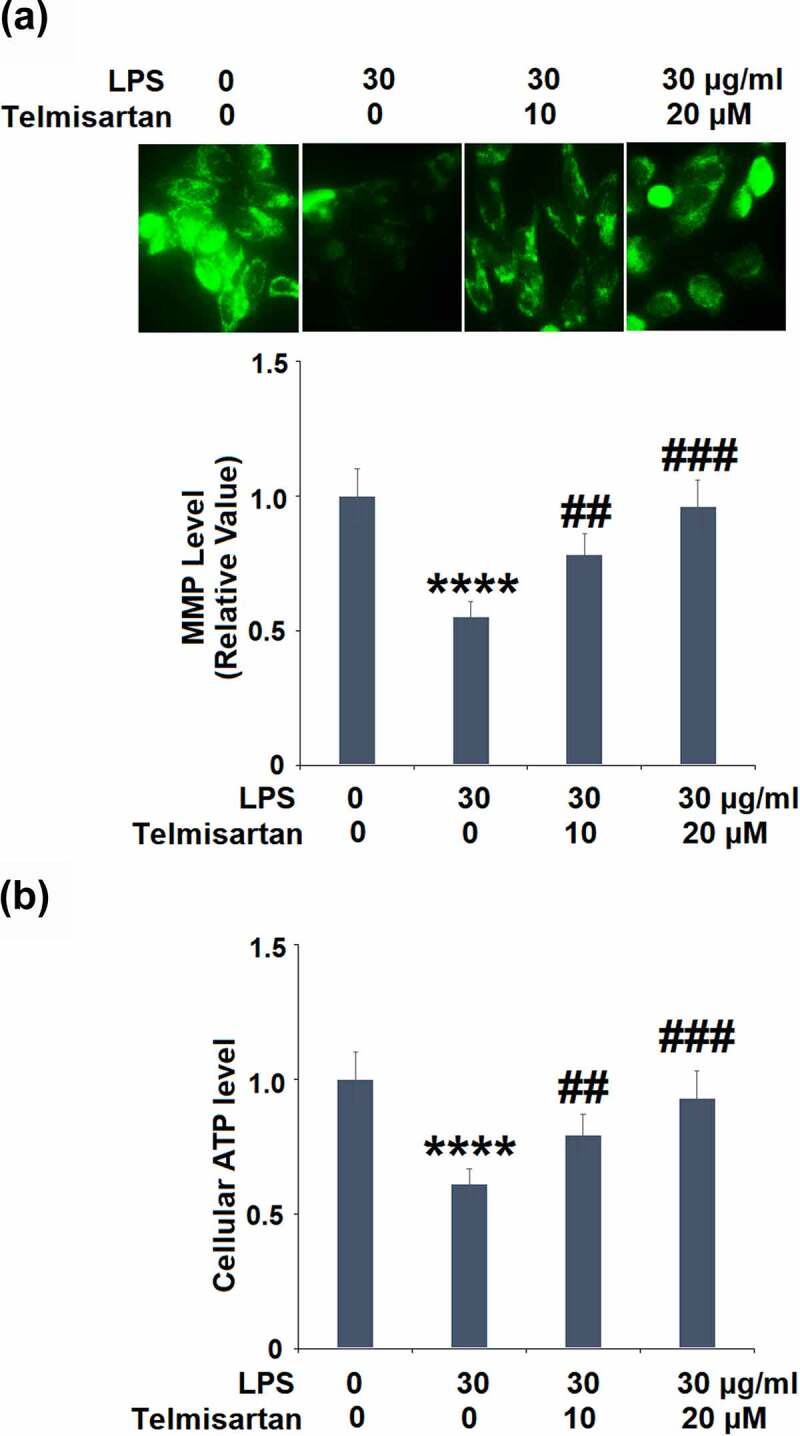
Telmisartan improved LPS-induced mitochondrial dysfunction in bronchial BEAS-2B epithelial cells. Cells were challenged with LPS (30 μg/ml) with or without Telmisartan (10, and 20 μM) for 24 hours. (a). Levels of mitochondrial membrane potential (ΔΨm) normalized to vehicle group; (b). level of cellular ATP normalized to vehicle group (****, P < 0.0001 vs. vehicle group; ##, ###, P < 0.01, 0.001 vs. LPS group)

Cells were challenged with LPS (30 μg/ml) with or without Telmisartan (10, and 20 μM) for 24 hours. As shown in Figure 2a, the level of mitochondrial membrane potential in the BEAS-2B epithelial cells was significantly inhibited by the stimulation of LPS but greatly elevated by treatment with Telmisartan in a dose-dependent manner. However, treatment with Telmisartan alone did not affect the levels of MMP (Data not shown). In addition, compared to the control, the level of cellular ATP (Figure 2b) was dramatically suppressed by stimulation with LPS but was extensively promoted by the treatment of Telmisartan. These data indicate that the mitochondrial dysfunction in bronchial BEAS-2B epithelial cells was significantly alleviated by treatment with Telmisartan.

### Telmisartan suppressed LPS-induced expression and production of proinflammatory cytokines

**Figure 3.**
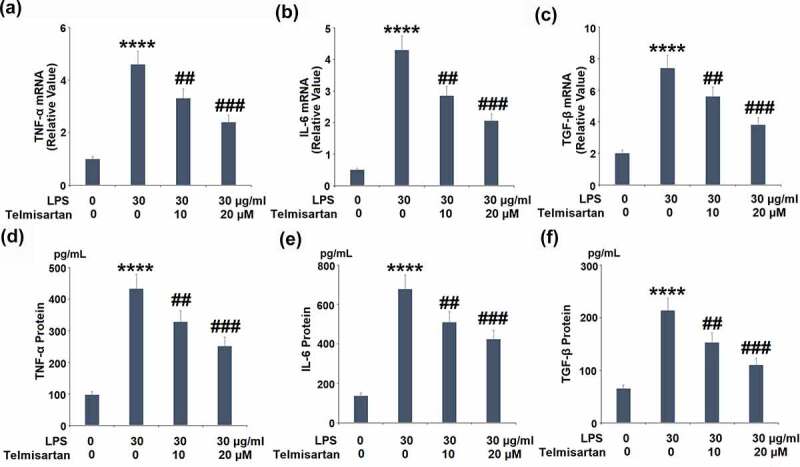
Telmisartan suppressed LPS-induced expression and production of proinflammatory cytokines. Cells were challenged with LPS (30 μg/ml) with or without Telmisartan (10, and 20 μM) for 24 hours. (a-c). mRNA of TNF-α, IL-6, and TGF-β normalized to control group; (d-f). Production of TNF-α, IL-6, and TGF-β (****, P < 0.0001 vs. vehicle group; ##, ###, P < 0.01, 0.001 vs. LPS group)

We further detected the concentration of released inflammatory factors in the treated BEAS-2B epithelial cells. As shown in Figure 3a–c, the gene expressions of TNF-α, IL-6, and TGF-β were significantly elevated by stimulation with LPS but pronouncedly suppressed by treatment with Telmisartan. As shown in Figure 3d–e, compared to control, the production of TNF-α by BEAS-2B epithelial cells was elevated from 96.7 to 433.6 pg/mL by stimulation with LPS, but significantly suppressed to 328.5 and 251.4 pg/mL by the administration of 10, and 20 μM Telmisartan, respectively. In addition, the concentrations of IL-6 in the control, LPS, 10, and 20 μM Telmisartan groups were 136.7, 678.3, 510.5, and 423.7 pg/mL, respectively. Lastly, compared to control, the secretion of TGF-β by BEAS-2B epithelial cells was promoted from 65.2 to 213.7 pg/mL by stimulation with LPS, but dramatically decreased to 152.5 and 109.8 pg/mL by the treatment of 10, and 20 μM Telmisartan, respectively. These data indicate that the severe inflammation in BEAS-2B epithelial cells induced by LPS was significantly ameliorated by Telmisartan.

### Telmisartan suppressed LPS-induced production of MUC5AC

**Figure 4.**
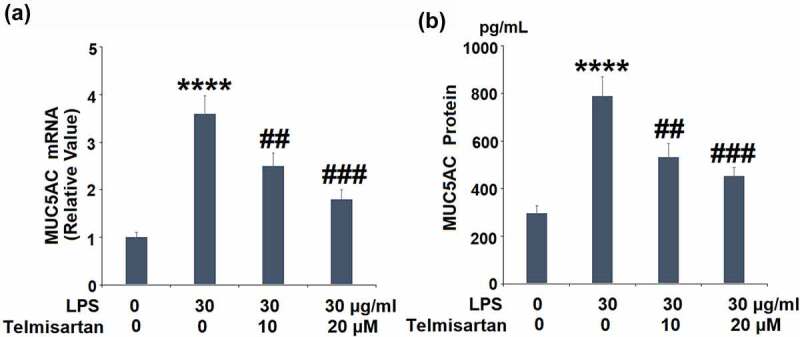
Telmisartan suppressed LPS-induced production of MUC5AC. Cells were challenged with LPS (30 μg/ml) with or without Telmisartan (10, and 20 μM) for 24 hours. (a). mRNA of MUC5AC normalized to vehicle group; (b). Production of MUC5AC (****, P < 0.0001 vs. vehicle group; ##, ###, P < 0.01, 0.001 vs. LPS group)

We further measured the expression of MUC5AC released by the BEAS-2B epithelial cells. As shown in Figure 4a, the gene expression of MUC5AC was significantly elevated by stimulation with LPS, but suppressed by the introduction of Telmisartan. In addition, compared to the control, the production of MUC5AC was extensively promoted from 296.1 to 789.5 pg/mL by stimulation with LPS, but inhibited to 533.2 and 426.8 pg/mL by treatment with 10, and 20 μM Telmisartan, respectively. These data indicate that the excessive expression level of MUC5AC induced by LPS was dramatically suppressed by Telmisartan (Figure 4b).

### The protective effects of Telmisartan against LPS-induced production of MUC5AC are mediated by the SOCS1/NF-κB signaling

**Figure 5.**
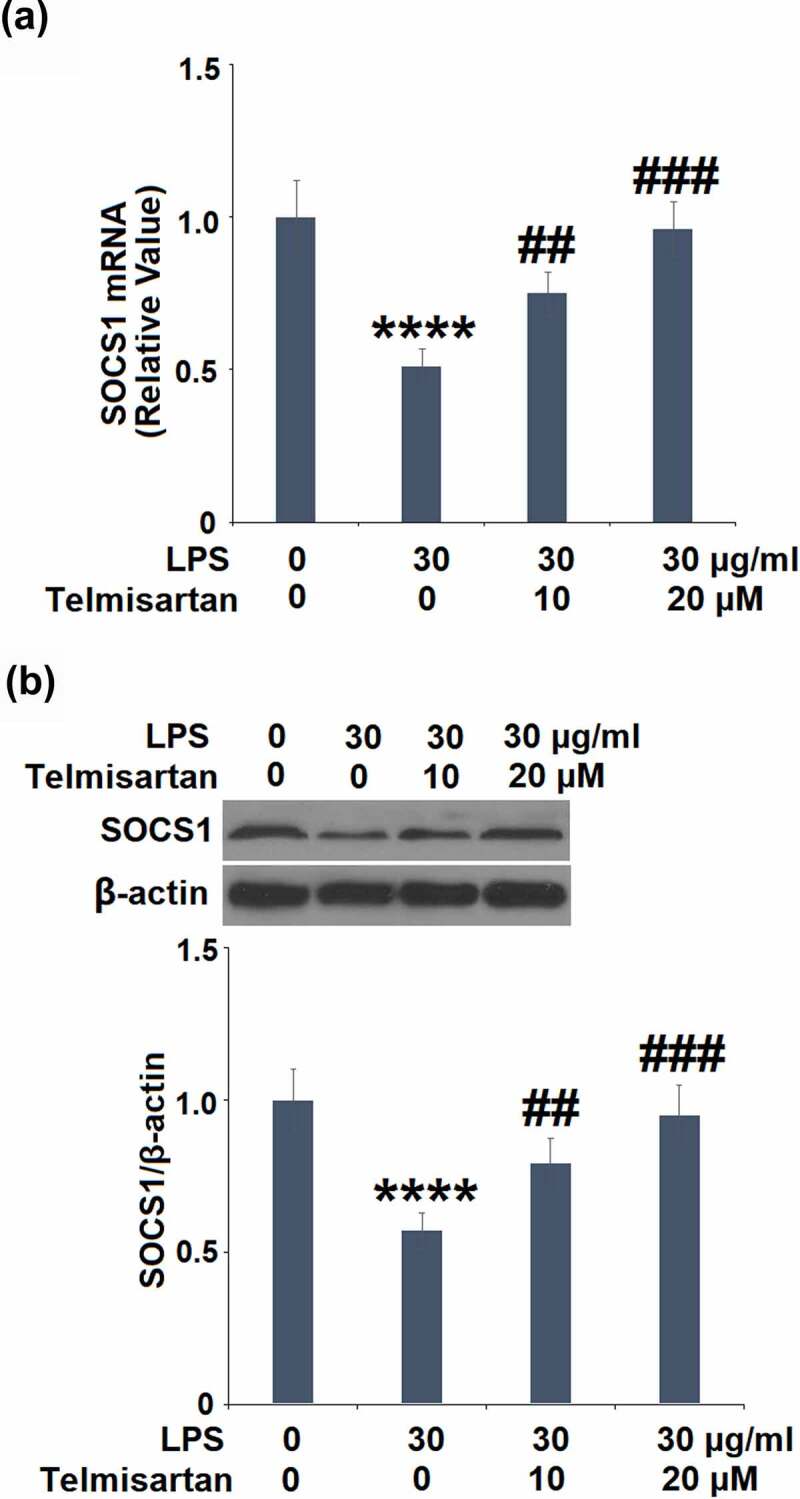
Telmisartan alleviated LPS-induced decrease in the expression of SOCS1. Cells were challenged with LPS (30 μg/ml) with or without Telmisartan (10, and 20 μM) for 24 hours. (a). mRNA of SOCS1 normalized to vehicle group; (b). Protein of SOCS1 normalized to vehicle group (****, P < 0.0001 vs. vehicle group; ##, ###, P < 0.01, 0.001 vs. LPS group)

To explore the mechanism underlying the regulatory effect of Telmisartan on the production of MUC5AC, the expression of SOCS1 in BEAS-2B epithelial cells was determined. Treatment with 10, and 20 μM Telmisartan alone increased SOCS1 mRNA and protein expressions (Data not shown). As shown in Figure 5, we found that the reduced expression level of SOCS1 induced by LPS was significantly upregulated by the administration of Telmisartan.

**Figure 6.**
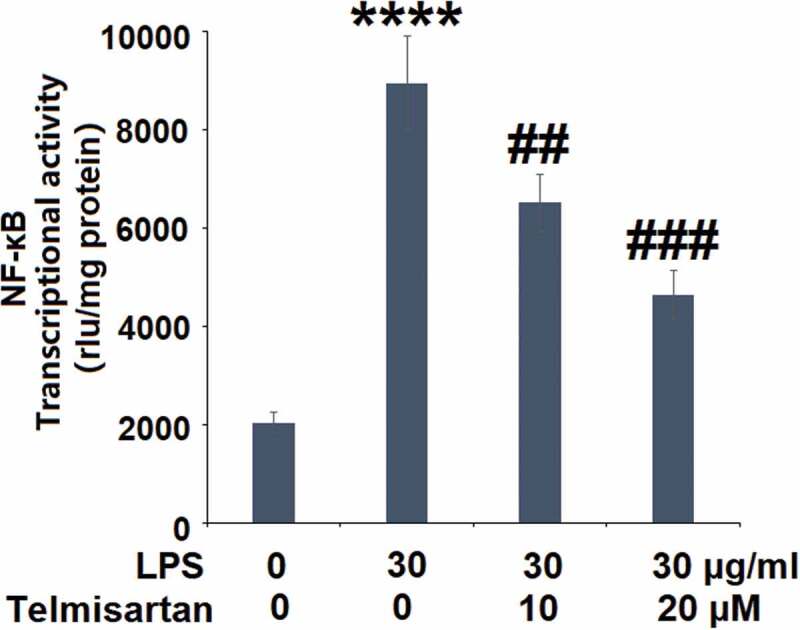
Telmisartan inhibited LPS-induced activation of NF-κB. Cells were transfected with NF-κB dependent luciferase expression plasmid, followed by stimulation with LPS (30 μg/ml) with or without Telmisartan (10, and 20 μM) for 24 hours. Luciferase activity was measured (****, P < 0.0001 vs. vehicle group; ##, ###, P < 0.01, 0.001 vs. LPS group)

Using a NF-κB-dependent luciferase reporter assay, the transcriptional activity of NF-κB following LPS stimulation and Telmisartan treatment was determined. Interestingly, the luciferase activity assay in Figure 6 demonstrates that exposure to LPS significantly increased the transcriptional activity of NF-κB, which was attenuated by Telmisartan dose-dependently.

**Figure 7.**
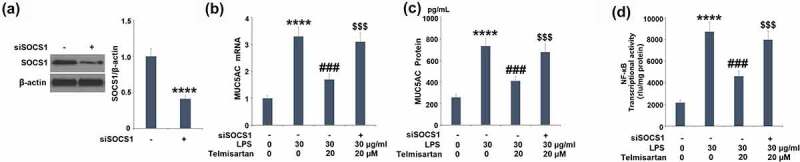
Silencing of SOCS1 abolished the protective effects of Telmisartan against LPS- induced production of MUC5AC. Cells were transfected with SOCS1 siRNA, followed by challenging with LPS (30 μg/ml) with or without Telmisartan (10, and 20 μM) for 24 hours. (a). Western blot analysis revealed successful knockdown of SOCS1; (b). mRNA of MUC5AC; (c). Production of MUC5AC; (d) Transcriptional activity of NF-kB measured by a luciferase assay (****, P < 0.0001 vs. vehicle group; ###, P < 0.001 vs. LPS group; $$$, P < 0.001 vs. LPS+ Telmisartan group)

To verify the involvement of upregulated SOCS1 in the regulatory effect of Telmisartan on the production of MUC5AC, cells were transfected with SOCS1 siRNA, followed by challenge with LPS with or without Telmisartan (20 μM) for 24 hours. As shown in Figure 7a, the expression level of SOCS1 was significantly inhibited by the transfection of SOCS1 siRNA. As shown in Figure 7b, the elevated gene expression of MUC5AC was significantly suppressed by introducing Telmisartan, it was reversed by the transfection of SOCS1 siRNA. In addition, compared to the LPS group, the production of MUC5AC was suppressed from 733.1 to 409.5 pg/mL by introducing Telmisartan, and then promoted to 677.3 pg/mL by the transfection of SOCS1 siRNA (Figure 7c). Notably, luciferase activity in Figure 7d demonstrates that silencing of SOCS1 abolished the inhibitory effects of Telmisartan against LPS-induced increase in transcriptional activity of NF-κB. These data indicate that the protective effects of Telmisartan against LPS-induced production of MUC5AC were significantly reversed by silencing of SOCS1.

## Discussion

Airway mucus is a layer of mucus at the bottom of the epithelium in the normal airway at a thickness of 5 to 50 microns, it blocks the toxins and pathogens from infiltrating the lung tissues and removes the infectious factors in the airway. Therefore, airway mucus is regarded as the first line of defense in respiratory immunity [20]. Under the pathological state, the airway mucus is excessively accumulated and is challenging to remove, contributing to airway stenosis or even airway occlusion [21,22]. Mucin is a kind of glycosylated glycoprotein with high molecular weights and is mainly produced by airway epithelial cells, it possesses the polypeptide skeleton of serine and threonine [23]. Mucin affects the properties of cilia and airway mucus and induces its viscoelasticity of airway mucus [24], involved in defense against external bioactive substances, epithelial growth, cell recognition, and signal transduction. The mucin production can be impacted by multiple factors, such as inflammatory factors, infectious elements, and immune factors, at a transcriptional or post-transcriptional level [25,26]. MUC5AC is a kind of gel-forming mucin and is reported to be up-regulated when inflammation develops in the airway and is closely related to the pathogenesis of asthma and chronic obstructive pulmonary disease [27,28]. Its expression of can be regulated by the released inflammatory factors in the bronchial goblet cells [29]. Two forms of mucin are highly expressed in human airways: *MUC5AC* and *MUC5B*. In an animal study, genetic disruption of MUC5B is required for airway clearance [30]. However, MUC5B is mainly expressed in the distal lungs and performs baseline barrier and clearance functions. However, MUC5AC production is greater than MUC5B in proximal airways, suggesting that MUC5AC could play a vital role. In COPD, MUC5AC was reduced but MUC5B was not affected, suggesting that both MUC5AC and MUC5B play a role in airway regulation [31].

LPS has been widely used to establish ALI models *in-vitro* and *in-vivo*, it could induce a severe inflammatory response and pathological injury [32]. Here, we report that the excessive production of inflammatory factors was accompanied by the upregulation of MUC5AC, which was significantly suppressed by the treatment of Telmisartan, indicating that the protective effect of Telmisartan against LPS-induced injury on bronchial BEAS-2B epithelial cells was related to the downregulation of MUC5AC. In our future work, the protective effect of Telmisartan against ALI will be further investigated by establishing the LPS-induced ALI animal model to further confirm the *in vivo* therapeutic effect of Telmisartan. Mitochondrial dysfunction in lung epithelial cells plays an important role in the pathogenesis of various lung diseases including ALI [33]. Mitochondria in epithelial cells control cellular energy metabolism and maintain homeostasis. Their malfunction could cause cellular stress or injury [34]. Mitochondrial membrane potential (MMP) is an essential indicator of mitochondrial function. The amelioration of reduced MMP indicates the effect of Telmisartan on bronchial epithelium which could modulate metabolic response and other energy utilization processes.

Suppressors of cytokine signaling (SOCS) are a group of immunosuppressants, including SOCS1-7 [35]. As the negative regulators that block the signaling transduction of cytokines, SOCS are involved in the pathogenesis of multiple diseases induced by inflammation, they directly inhibit the activity of Janus kinase (JAK) and the subsequent activity of signal transduction and transcriptional activators (STAT) [36]. SOCS1 interacts with the ubiquitin ligase system to induce the degradation of target proteins, such as NF-κB and the Jun-N terminal kinase pathway [37], further regulating the expression of inflammatory factors. Recently, SOCS1 has been reported to regulate the expression of MUC5AC [16]. In the present study, we found that the expression level of SOCS1 was significantly elevated by treatment with Telmisartan, indicating an activating effect of Telmisartan on the negative regulatory pathway of inflammation.

Further investigations revealed that the protective effects of Telmisartan against LPS-induced induction of MUC5AC were significantly abolished by silencing SOCS1, indicating that the regulatory effect of Telmisartan on the expression of MUC5AC was related to the upregulation of SOCS1. A previous study demonstrated that SOCS1 is the suppressor of cytokine-induced hyper-inflammation [15,16]. We hypothesize that the increase in SOCS1 expression by Telmisartan leads to reduced cytokine production. In our future work, the effect of Telmisartan on the upstream cascades of SOCS1 will be further investigated to better understand the regulatory effect of Telmisartan on SOCS1. Interestingly, type-1 and type-2 angiotensin receptors and peroxisome proliferator-activated receptor γ (PPARγ) have been reported to be expressed in bronchial epithelial cells and play an important role in regulating inflammation and mucin expression in allergic airway disease [38,39]. Therefore, the direct target of Telmisartan will be explored to figure out the mechanism underlying the interaction between Telmisartan and SOCS1.

## Conclusion

This study highlights the novel role of Telmisartan in mitigating LPS-induced damage to human bronchial epithelial cells by improving mitochondrial function and inhibiting the expressions of pro-inflammatory cytokines. Notably, Telmisartan ameliorated LPS-induced production of MUC5AC through the SOCS1/NF-κB axis.
